# Silver-Doped Calcium Phosphate Bone Cements with Antibacterial Properties

**DOI:** 10.3390/jfb7020010

**Published:** 2016-04-18

**Authors:** J. V. Rau, M. Fosca, V. Graziani, A. A. Egorov, Yu. V. Zobkov, A. Yu. Fedotov, M. Ortenzi, R. Caminiti, A. E. Baranchikov, V. S. Komlev

**Affiliations:** 1 Istituto di Struttura della Materia, Consiglio Nazionale delle Ricerche, CNR-ISM, Via del Fosso del Cavaliere 100, Rome 00133, Italy; marco.fosca@ism.cnr.it (M.F.); valerio.graziani@artov.ism.cnr.it (V.G.); marco.ortenzi@ism.cnr.it (M.O.); 2 A.A. Baikov Institute of Metallurgy and Materials Science, Russian Academy of Sciences, Leninsky Prospect 49, Moscow 119334, Russia; alex1814@yandex.ru (A.A.E.); zyv13@mail.ru (Y.V.Z.); fedoto_ayu@mail.ru (A.Y.F.); 3 Dipartimento di Chimica, Università di Roma “La Sapienza”, Piazzale Aldo Moro 5, Rome 00185, Italy; ruggero.caminiti@uniroma1.it; 4 Kurnakov Institute of General and Inorganic Chemistry of the Russian Academy of Sciences, Leninsky Prospect 31, Moscow 119991, Russia; a.baranchikov@yandex.ru

**Keywords:** bone cement, calcium phosphate, tricalcium phosphate, silver, CaAg(PO_3_)_3_, infection, bone graft

## Abstract

Calcium phosphate bone cements (CPCs) with antibacterial properties are demanded for clinical applications. In this study, we demonstrated the use of a relatively simple processing route based on preparation of silver-doped CPCs (CPCs-Ag) through the preparation of solid dispersed active powder phase. Real-time monitoring of structural transformations and kinetics of several CPCs-Ag formulations (Ag = 0 wt %, 0.6 wt % and 1.0 wt %) was performed by the Energy Dispersive X-ray Diffraction technique. The partial conversion of β-tricalcium phosphate (TCP) phase into the dicalcium phosphate dihydrate (DCPD) took place in all the investigated cement systems. In the pristine cement powders, Ag in its metallic form was found, whereas for CPC-Ag 0.6 wt % and CPC-Ag 1.0 wt % cements, CaAg(PO_3_)_3_ was detected and Ag (met.) was no longer present. The CPC-Ag 0 wt % cement exhibited a compressive strength of 6.5 ± 1.0 MPa, whereas for the doped cements (CPC-Ag 0.6 wt % and CPC-Ag 1.0 wt %) the reduced values of the compressive strength 4.0 ± 1.0 and 1.5 ± 1.0 MPa, respectively, were detected. Silver-ion release from CPC-Ag 0.6 wt % and CPC-Ag 1.0 wt % cements, measured by the Atomic Emission Spectroscopy, corresponds to the average values of 25 µg/L and 43 µg/L, respectively, rising a plateau after 15 days. The results of the antibacterial test proved the inhibitory effect towards pathogenic *Escherichia coli* for both CPC-Ag 0.6 wt % and CPC-Ag 1.0 wt % cements, better performances being observed for the cement with a higher Ag-content.

## 1. Introduction

Calcium phosphate bone cements (CPCs) are widely used for bone graft substitution due to their chemical similarity to the bone mineral part. CPCs have excellent biological behavior and they can be applied as injectable and biodegradable grafting materials.

However, despite a number of publications on the development of CPCs for bone replacement and tissue engineering in the last decades [1], there is still a need for their functionalization. One of the major problems, related to the bone graft applications, is linked with infections, this issue remaining open [2]. Bacterial infections associated with the introduction of materials into the damaged part and/or bone defects lead to inflammations, eventually resulting in bone loss [3]. As a consequence, an increase of the cost treatment due to the additional surgery is required for the rehabilitation and extended recovery of patients [4,5]. The use of antibiotics in the bone graft or oral applications before surgery does not provide sufficient protection. In fact, the wrong antibiotics or low doses can create resistant strains of bacteria, which are difficult to treat afterwards [6,7,8].

The bone graft associated infections may be prevented by doping of synthetic bone grafting materials with suitable metal ions (e.g., Ag^+^) at low (non-cytotoxic) concentrations [9,10]. This way seems to be more appropriate, because the antimicrobial activity could be provided directly at the implantation site (target delivery). The effects of Ag^+^ on microorganisms are well known and reported elsewhere [11,12].

In this study, we demonstrated the use of a relatively simple processing route based on preparation of silver-doped CPCs (CPCs-Ag) through the preparation of Ag-containing solid dispersed active phase. Further, the real-time monitoring of structural transformations and kinetics of several CPCs-Ag formulations based on β-tricalcium phosphate (β-TCP) (CPC-Ag 0 wt %, CPC-Ag 0.6 wt % and CPC-Ag 1.0 wt %) was carried out. The formation of new phases was followed *in situ* by the Energy Dispersive X-ray Diffraction technique (EDXRD), allowing one to obtain a 3D map of diffraction patterns, collected as a function of the scattering parameter and of time. This technique proved to be suitable to study the real-time monitoring of the CPCs hardening process *in situ* [13,14,15]. The EDXRD structural investigations were complemented with the Scanning Electron Microscopy (SEM) morphological studies, compressive strength measurements and the Ag^+^ release monitoring by the Atomic Emission Spectroscopy (AES). The antibacterial *in vitro* test, using pathogenic *Escherichia coli*, was performed to prove the inhibitory effect of the Ag-containing cement formulations.

## 2. Experimental Section

### 2.1. Synthesis of Silver-Doped Tricalcium Phosphate Powders

Silver-doped tricalcium phosphate powders as solid phase of CPCs were synthesized via precipitation from aqueous solutions at 25 °C. Briefly, 940 or 910 mL of 0.5 M solutions of calcium nitrate (CAS 13477-34-4, Sigma, Dorset, UK) and 58.9 or 88.3 mL of 0.1 M solutions silver nitrate (CAS 7761-88-8, Sigma, Dorset, UK) (the total volume of the salt mixture must be 1000 mL) were mixed with 667 mL of 0.5 M (NH_4_)_2_HPO_4_ (CAS 7783-28-0, Sigma, Dorset, UK) dropwise during 10 min. The pH value of this system was kept at around 7 by adding 150 ± 20 mL 25% aqueous ammonia solution. Afterwards, the suspension was mixed for 2 h. After that, the precipitate was separated on a Buchner funnel and dried at 80 °C and then sintered in the air at 1300 °C for 2 h. The resulting powders were ground in ethanol and dried at 60 °C.

Pure TCP powder, used for control cement samples, was produced by the same procedure described above, without using the silver nitrate. The calcination of powder at 850 °C for 2 h was performed.

### 2.2. Hardening Liquid Preparation

1.5 M solution of magnesium dihydrogen phosphate tetrahydrate (CAS 13092-66-5, Alfa Chemistry, Holtsville, NY, USA) with 0.1 M phosphoric acid (CAS 7664-38-2, Sigma, Dorset, UK) and 30 wt % glycerol solution (CAS 56-81-5, Sigma, Dorset, UK) were used as hardening liquid (HL).

### 2.3. Preparation of CPCs-Ag Specimens

CPC-Ag powders and hardening liquid were mixed in a powder-to-liquid ratio (P/L) 4/3 (e.g., 1 g of CPC-Ag powder and 0.75 g of hardening liquid) in a glass mortar for approximately 180 s. The pastes were then poured into cylindrical Teflon molds, 15 mm or 6 mm in diameter and 10 mm or 12 mm in depth, respectively, which were stored at either 20 °C or 37 °C and kept on air and in distilled water. The obtained CPC-Ag specimens were used for further analyses.

### 2.4. Setting Time Measurements

The setting times of the CPC-Ag pastes were evaluated using the tip (1 mm diameter) of a Vicat needle with a 400 g load (according to the ISO standard 9917) to make a perceptible circular indentation on the cement surface. The CPC-Ag pastes were filled into cylindrical Teflon molds of 15 mm diameter and 10 mm depth. Each sample was incubated in a humidor with 100% relative humidity at 37 °C for setting time measurements.

### 2.5. pH Measurements

The pH measurements were performed as follows: samples of crushed cement (1 g) after hardening were placed into a 50 mL volume flask and distilled water was added up to the 50 mL volume. The pH value was measured after 0.5, 1, 3, 5 and 24 h of soaking time and at 37 °C, using an Econix-Expert 001 pH meter ((Econix-Expert Ltd, Moscow, Russia).

### 2.6. Energy Dispersive X-ray Diffraction

The Energy Dispersive X-ray Diffraction technique is based on the use of a non-commercial apparatus, the EDXRD diffractometer [16]. It consists of white X-ray radiation that is produced by a commercial W-anode X-ray tube (up to 50 keV) and a solid-state detector, in our case an EG&G high purity Germanium photodiode, whose energy resolution is about 1.5%–2.0% in the 20–50 keV energy range.

The detector is connected to a PC via the ADCAM hardware and the signal is processed by a Maestro software, which performs the necessary analog to digital conversions. These components are located at the end of the two arms pivoting around the optical center of the device. In this way, the reciprocal space scan necessary to collect the diffraction pattern, is carried out electronically, rather than mechanically, as in the conventional Angular Dispersive X-ray Diffraction method. Diffraction patterns, collected in this way, represent the diffracted intensity (*n*° of incident X-photons) as a function of scattering parameter *q* (*q* = *aE*sinϑ), where *q* is the normalized momentum transfer magnitude, *a* is a constant, *E* is the energy of the incident X-ray beam and 2ϑ is the scattering angle). With the scan being carried out electronically, the experimental geometry can be kept fixed during pattern acquisition. This allows a faster recording of the Bragg peaks, since in the ED mode, the whole diffraction pattern is obtained in parallel at any q value.

As preliminary tests for powder samples, several diffraction patterns were collected *ex situ* at various scattering angles, in order to find the best experimental conditions. All the measurements were performed with a scattering angle of 2ϑ = 10°, energy of 50 keV and current of 30 mA.

For *in situ* EDXRD measurements, immediately after mixing of cement powder and hardening liquid, cement paste was quickly placed in the optical center of the diffractometer. Collection of the diffraction patterns proceeded at high rate during the first 30 min of the experiment (one spectrum every 2 min) and, afterwards, every 15 min until the end of the experiment. The experiments were performed at room temperature (20 °C).

### 2.7. Scanning Electron Microscopy

The morphology of samples was investigated using a Carl Zeiss NVision 40 high resolution Scanning Electron Microscope, equipped with an Oxford Instruments X-Max energy dispersive detector (80 mm^2^) (Carl Zeiss Inc., Oberkochen, Germany). The CPC-Ag cement samples were investigated after incubation in a humidor with 100% relative humidity at 37 °C and at 24 h after the end of mixing. The images were obtained at 1 kV acceleration voltage (SE2, magnifications up to ×100 k). The CPC-Ag cement samples were analyzed without deposition of a conductive layer on their surface.

### 2.8. Compressive Strength Measurements

The compressive strength was evaluated according to the ISO standard 9917. The investigated samples, immediately after the preparation, were immersed in distilled water grade 3, as defined in the ISO standard 3696:1987, at (37 ± 1) °C for (23 ± 0.5) h. The prepared cylindrical samples (about 12 mm of height and 6 mm of diameter) were tested at 24 h after the end of mixing (five samples for each type of cement). Compression tests were carried out using an Instron 4082 (Instron Pty. Ltd., Buckinghamshire, UK) machine, operating at a crosshead speed of 1 mm·min^−1^. Statistical analysis was performed using the SPSS software, version 17.0 (Statistical Package for Social Sciences, SPSS Inc., New York, NY, USA). The mean and standard deviation values of compressive strength were calculated.

### 2.9. Silver-Ion Release

Silver-ion release from the cement samples was studied in TRIS-HCl buffer solution at pH 7.4 for 30 days at a constant liquid phase volume (closed system). The buffer solution was adjusted to pH = 7.4 by adding 13.25 g of TRIS (Cat. No.: 77-86-1, Sigma-Aldrich, Dorset, UK) and 125 mL of HCl (Cat. No.: 7647-01-0, Aldrich-Aldrich, Dorset, UK). The silver-ion release measurements were performed for crushed cements with the solid-to-liquid ratio of 0.5 g/100 mL and at 37 °C. At least three samples (the solid-to-liquid) were performed and were investigated for each time points (1, 3, 7, 14, and 28 days). The silver concentration in the liquid phase was measured using an Atomic Emission Spectrometer Ultima 2 (Jobin-Yvon, Longjumeau, France).

### 2.10. Antibacterial Test

Cylindrical samples (about 10 mm of height and 15 mm of diameter) with smooth surfaces were prepared for three cement systems (CPC-Ag 0 wt % (control sample), CPC-Ag 0.6 wt % and CPC-Ag 1.0 wt %). The tested pathogenic bacteria strain was *Escherichia coli* (K-12 substr. MG1655). The microorganisms were grown in LB broth at 37 °C. For each cement disk, the inoculation density of bacteria was kept constant in each of the three used Petri dishes, in order to assure conditions for experimental repeatability. An inoculum was prepared in liquid medium, corresponding to a turbidity of 0.5 (≈106 UFC/mL) on the McFarland standard. The culture medium used for testing was agar. The incubation period was 18–24 h. The plates were read after 24 h and 48 h. All experiments were carried out in triplicate and the reported data represent average values ±SD.

## 3. Results and Discussion

**Figure 1:**
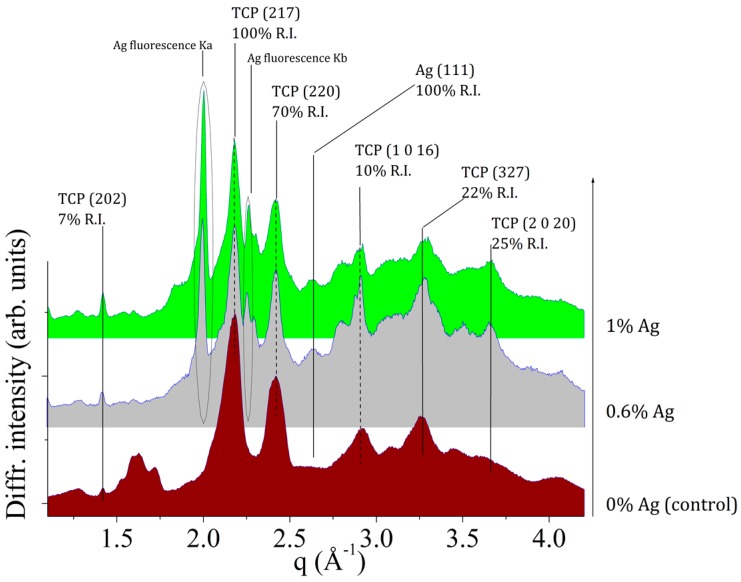
EDXRD spectra of pristine powder samples: CPC-Ag 0 wt % (control), CPC-Ag 0.6 wt %, and CPC-Ag 1.0 wt %.

For structural characterization of the pristine powder samples (CPC-Ag 0 wt %, CPC-Ag 0.6 wt % and CPC-Ag 1.0 wt %) *ex situ* EDXRD measurements were performed and the results of powder characterization are shown in Figure 1. The main phase was identified as β-TCP (card number 70-2065 [17]) for all the collected patterns, the most intense peaks falling in the q-range of 1.0–4.0 Å^−1^. Furthermore, in the case of CPC-Ag 0.6 wt % and CPC-Ag 1.0 wt % powder samples, additional peaks were detected. In particular, the presence of metallic Ag is proved by the diffraction peak located at *q* = 2.6 Å^−1^, attributed to the Ag (met.), ((111), 100% R.I.) (card number 87-0720 [17]), supported by the Ag Kα and Kβ fluorescence peaks presence, at energy values of 21.0 keV and 22.16 keV, respectively. An accurate peak attribution did not evidence any reflections belonging to other Ag-compounds for CPC-Ag 0.6 wt % and CPC-Ag 1.0 wt % powders.

It is well known that the most common basic calcium source in dicalcium phosphate dihydrate (DCPD) cement systems is tricalcium phosphate. This alkaline calcium phosphate has two crystallographic forms β- and α-TCP, and both minerals have been used to prepare DCPD cements [1]. On the other hand, authors [18] reported silver-doped calcium phosphate with the Ag^+^ ions incorporated into calcium phosphate lattice and that its heating at 1300 °C led to TCP with almost no silver content. This means that at such a high temperature it is not possible to obtain solid dispersed active phase containing Ag^+^ for the DCPD cement systems. Taking into account the reported literature data, in our work, the pH value was adjusted to 7 during the chemical reaction, and as a result a powder, consisting of poorly crystalline apatite and silver phosphate, was obtained. The calcination of such silver-doped calcium phosphate powders at 1300 °C led to β-TCP with the presence of Ag (met.), due to the silver phosphate decomposition (see the X-ray diffraction data presented in Figure 1). Our results also indicate that Ag presence stabilizes β-TCP lattice at 1300 °C, because otherwise, without Ag at this temperature, α-TCP is formed.

In order to follow phase transformations taking place when the pristine powders are mixed with hardening liquid, *in situ* time-resolved EDXRD measurements were carried out.

**Figure 2:**
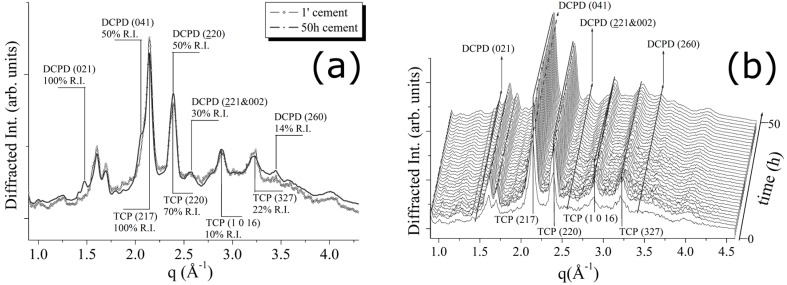
(**a**) Comparison between the first (after 1 min) and the last (after 50 h) diffraction pattern obtained upon CPC-Ag 0 wt % (control) cement; (**b**) 3D map of diffraction patterns collected to follow CPC-Ag 0 wt % (control) cement hardening process.

For the CPC-Ag 0 wt % (control) cement system, the transformations occurring during the cement paste hardening were monitored, by collecting the EDXRD spectra for a total time of 50 h (see Figure 2). In Figure 2a, a comparison between the first and the last EDXRD pattern, collected upon the CPC-Ag 0 wt % cement after 1 min (black line) and after 50 h (grey line) of the hardening process, respectively, is shown.

In Figure 2b, a 3D perspective of a sequence of spectra collected during 50 h of hardening process is reported. As can be observed, some differences are present. Indeed, new reflections appeared, while the existing peaks increased their intensities. After an accurate attribution, the new phase was identified as dicalcium phosphate dihydrate, DCPD (Brushite, CaHPO_4_·2H_2_O). New reflections, labelled as DCPD (021) (100% R.I.), (041) (75% R.I.) and (221 & 022) (30% R.I.) (card number 09-0077 [17]), testify the partial conversion of the β-TCP phase into the DCPD. In particular, an increase of the β-TCP (220) peak intensity was registered (see Figure 2), whereas other β-TCP (220) and β-TCP (327) peak intensities can be considered unchanged or even slightly decreased. Since not all the peaks, attributed to the β-TCP phase, behaved in a similar way, the registered changes cannot be ascribed to the β-TCP crystallization, but rather to the appearance of the new DCPD phase, DCPD (220) reflection sharing the same q-position with the β-TCP (220) peak.

**Figure 3:**
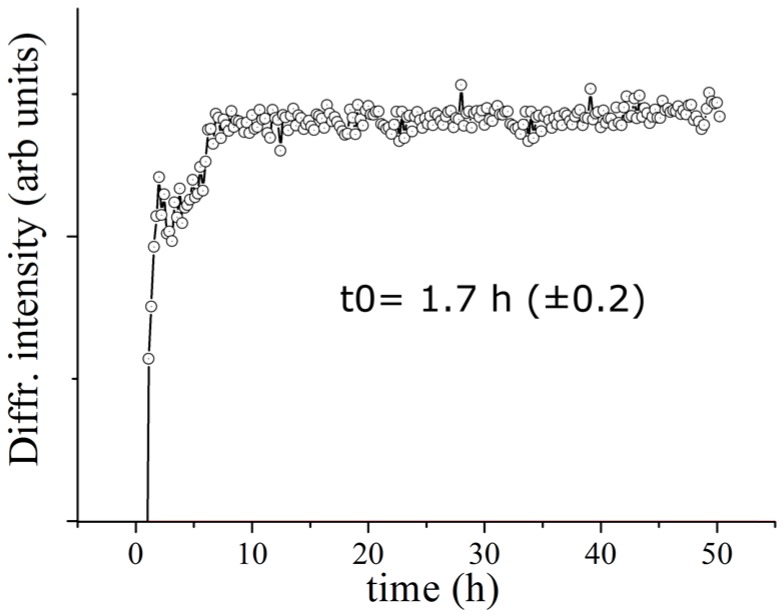
Diffracted DCPD (041) Bragg reflection intensity *versus* time (CPC-Ag 0 wt % cement system).

In order to estimate the characteristic transformation time of the evolving system, a quantitative analysis was performed for the DCPD (041) Bragg reflection, as representative for the DCPD phase. A Gaussian fit procedure was carried out upon each collected pattern. The plot of diffracted intensity of the DCPD (041) Bragg’s reflections as a function of time is reported in Figure 3. Each point corresponds to the intensity of the Gaussian fit of the relative peak in the EDXRD spectrum. As can be seen the intensity ranges from an initial values very close to 0 up to the maximum values, defining a plateau region. Time evolution profile of the diffracted intensity shows a typical sigmoidal growth. Performing a sigmoidal fit, the characteristic time of the β-TCP transformation into DCPD was estimated to be 1.7 h.

**Figure 4:**
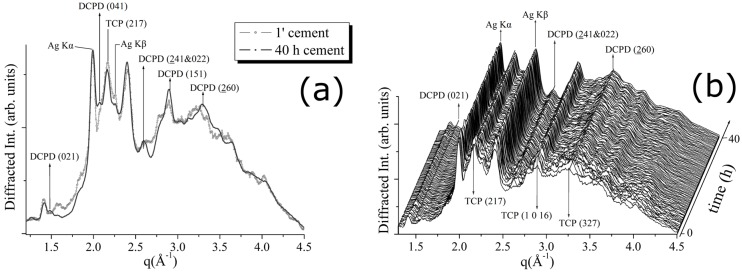
(**a**) Comparison between the first (after 1 min) and the last (after 40 h) diffraction pattern obtained upon CPC-Ag 0.6 wt % cement; (**b**) 3D map of diffraction patterns collected to follow CPC-Ag 0.6 wt % cement hardening process.

Similar diffraction data analysis was performed for the CPC-Ag 0.6 wt % cement system. Figure 4a shows a comparison between the pattern, collected after 1 min, and that collected after 40 h. In Figure 4b, a 3D perspective of a sequence of EDXRD spectra collected upon the CPC-Ag 0.6 wt % cement during 40 h of hardening process is reported. Also in this case, a conversion of the β-TCP into DCPD phase can be detected. Compared to the CPC-Ag 0 wt % (control) cement system (Figure 2 and Figure 3), the conversion rate is lower.

**Figure 5:**
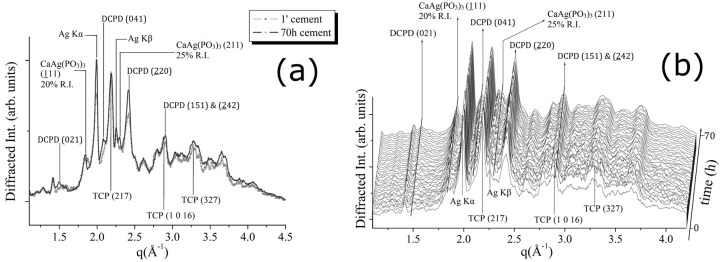
(**a**) Comparison between the first (after 1 min) and the last (after 70 h) diffraction pattern obtained upon CPC-Ag 1.0 wt % cement; (**b**) 3D map of diffraction patterns collected to follow CPC-Ag 1.0 wt % cement hardening process.

The experimental results obtained for the CPC-Ag 1.0 wt % cement system are shown in Figure 5. In Figure 5a, a comparison between the patterns, collected after 1 min and after 70 h of the hardening process, is shown. In Figure 5b, a 3D perspective of a sequence of EDXRD spectra collected upon the cement during 70 h of hardening process is reported. The appearance of new Bragg’s reflections (DCPD (021) (100% R.I.), DCPD (041) (75% R.I.)) takes place also in this case. This is in agreement with the changes observed in the previous two cement systems. Similarly, the β-TCP conversion into DCPD phase takes place, as testified by the increasing intensity of the DCPD (220) peak at *q* = 2.4 Å^−1^. The conversion rate is low, as well as for the CPC-Ag 0.6 wt % system, testified by the low intensities of the newly appeared peaks.

Unlike the previous two cement systems, for the CPC-Ag 1.0 wt % cement, the appearance of two new reflections at *q* = 1.64 (Å^−1^) and *q* = 2.3 (Å^−1^), accurately checked and confidently assigned to the new phase, CaAg(PO_3_)_3_ (card number 23-0126 [17]), (111) and (211) peaks, respectively, was detected (see Figure 5). Is it worth mentioning that the peaks belonging to this new phase become sharper at the late stage of the process (after about 50 h of hardening).

For the CPC-Ag 0.6 wt % cement, CaAg(PO_3_)_3_ peaks cannot be distinguished at *q* = 1.64 (Å^−1^) and *q* = 2.3 (Å^−1^) positions (see Figure 4), likely because the Ag amount is less (0.6 wt % compared to 1.0 wt %), and the background intensity is high. Despite of this, CaAg(PO_3_)_3_ presence also in the CPC-Ag 0.6 wt % cement could be indirectly testified by the presence of the Ag Kα and Kβ fluorescence peaks. It is important to stress here that Ag (met.) peaks are not present in the spectra of cement systems, but only in those of the powders.

Recently, calcium polyphosphates—novel materials for bone grafts, have been developed [19,20]. Divalent calcium-silver polyphosphate, CaAg(PO_3_)_3_, belongs to the group of inorganic polymers [21]. To our knowledge, there is no literature reporting the use of this compound as antibacterial agent.

**Figure 6:**
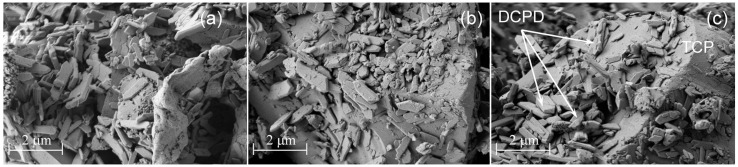
SEM images of (**a**) CPC-Ag 0 wt %; (**b**) CPC-Ag 0.6 wt % and (**c**) CPC-Ag 1.0 wt % cements after 24 h of hardening.

The SEM investigations confirm that after 24 h of hardening, the microstructure of the cement pastes for all three investigated cements was predominantly non homogeneous with the unreacted β-TCP particles and the newly formed DCPD phase, characterized by the lamellar morphology. In Figure 6, SEM images of CPC-Ag 0 wt %, CPC-Ag 0.6 wt % and CPC-Ag 1.0 wt % cements after 24 h of hardening are shown. As can be seen, the morphology of Ag-containing cement systems is pretty similar and two phases, *i.e.*, TCP and DCPD, can be well distinguished.

The setting time of the cement pastes was evaluated. The CPC-Ag 0.6 wt % and the CPC-Ag 1.0 wt % cements are characterised by the setting time of 5 and 7 min, respectively. For comparison, the CPC-Ag 0 wt % control cement has setting time of approximately 3 min. After setting, the pH value of all investigated cements was measured to be at physiological value (*i.e.*, 7.2–7.4).

**Figure 7:**
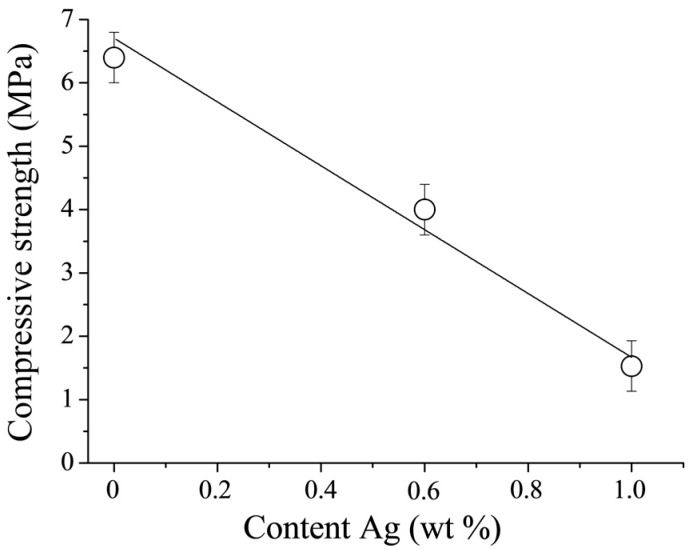
Compressive strength of the investigated cements at 24 h after the end of mixing.

The compressive strength of DCPD cements is known to be limited [1,22]. This is confirmed by the results of the compressive strength measurements performed in this work (Figure 7). The CPC-Ag 0 wt % cement exhibited a compressive strength of 6.5 ± 1.0 MPa, whereas upon doping with Ag, the mechanical characteristics change, revealing the reduced compressive strength of 4.0 ± 1.0 MPa and 1.5 ± 1.0 MPa for the CPC-Ag 0.6 wt % and CPC-Ag 1.0 wt % cements, respectively. It is reported in [23] that the introduction of Ag^+^ resulted in the increase of cement compressive strengths by approximately 30%, which is not in agreement with the present work, where the opposite effect, *i.e.*, the decreased compressive strength, was registered. In general, the strength of cement systems is strongly governed by phase composition. In our case, the strength properties are largely reduced with the increase of Ag content likely due to the formation of divalent calcium-silver polyphosphate in the final product of CPC-Ag 0.6 wt % and CPC-Ag 1.0 wt % cements.

**Figure 8:**
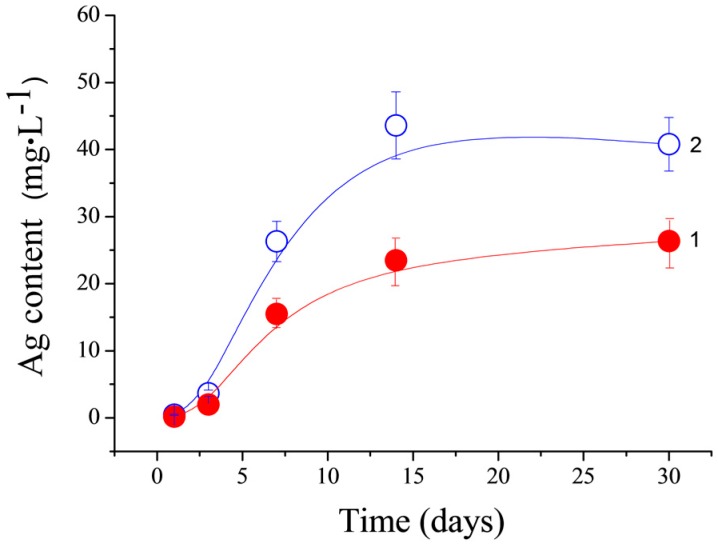
Silver-ion release from cement samples into TRIS-HCl buffer solution during 30 days of immersion (CPC-Ag 0.6 wt % (1) and CPC-Ag 1.0 wt % (2)).

The Ag^+^ release from the CPC-Ag 0.6 wt % and CPC-Ag 1.0 wt % cements was measured by the AES and the obtained results are shown in Figure 8 (circle and quadrate point lines, respectively). As expected for the CPC-Ag 1.0 wt % cement, the higher concentration of Ag^+^ is released. As can be seen, an increase up to 25 µg/L and 43 µg/L for the CPC-Ag 0.6 wt % and CPC-Ag 1.0 wt % cements, respectively, was detected, reaching a plateau after 15 days. The reached plateau can be an indication of the equilibrium established in the investigated systems. In this context, the toxicity threshold level of Ag^+^ should be considered for materials developed for clinical use [24], therapeutic/toxic effects of silver being a complex issue necessitating careful consideration of many factors, among them the silver state, the exposure to systematic long-term toxic concentrations, body mass, implant material mass, *etc.* Experimental studies suggest that Ag^+^ concentration of 60 ppm should be sufficient to control the majority of bacterial and fungal pathogens [25]. As to the eventual toxicity, roughly, for human use, the threshold value of silver level below 200 ppb in the blood is considered non-toxic [26]. Bone toxicity is still not widely recognised in the safety regulations of Ag and Ag-containing products, but recent clinical studies for soft tissues suggest an Ag “threshold” of about 100 µg/L in the blood [24]. The maximum amount released from our cements is 45 µg/L (corresponding to approximately 45 ppb), much below this threshold and, therefore, toxic effects are not expected. The Ag^+^ amount released from Ag-doped DCPD cements of about 30 µg/day was recently reported in [23].

**Figure 9:**
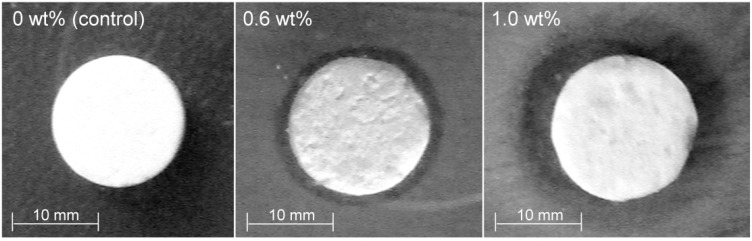
Inhibition zones for *Escherichia coli* at 48 h: disks are numbered according to their Ag^+^ content in CPCs specimens.

All three proposed cement formulations were tested for antibacterial properties. The reading of the agar plates was made at 24 h and 48 h. The inhibitory effect was proved by the formation of circular, clear zones of inhibition around the disks (Figure 9). Such effect was not observed for the control sample (CPC-Ag 0 wt %). The measured diameters of the inhibition zones are presented in Table 1. The inhibition zone diameter increased simultaneously with the increase of the Ag^+^ concentration in the samples. The best efficacy was obtained for the CPC-Ag 1.0 wt % cement. There were no significant differences between the readings at 24 h and 48 h of incubation time.

## 4. Conclusions

In this work, three cement systems were investigated: CPC-Ag 0 wt % (control sample), CPC-Ag 0.6 wt % and CPC-Ag 1.0 wt %. The structural changes taking place during the hardening process of the cements were followed by the EDXRD technique. The partial conversion of β-TCP phase into the DCPD took place in all three investigated cement systems. For Ag-containing cements (CPC-Ag 0.6 wt % and CPC-Ag 1.0 wt %) a lower conversion rate was observed.

In the pristine Ag-containing cement powders, Ag in its metallic form was registered, whereas in CPC-Ag 0.6 wt % and CPC-Ag 1.0 wt % cements systems, CaAg(PO_3_)_3_ was present and Ag (met.) was no more detectable.

The CPC-Ag 0 wt % cement exhibited a compressive strength of 6.5 ± 1.0 MPa, whereas for the doped cements (CPC-Ag 0.6 wt % and the CPC-Ag 1.0 wt %) reduced values of the compressive strength 4.0 ± 1.0 MPa and 1.5 ± 1.0 MPa, respectively, were registered.

Ag^+^ release from CPC-Ag 0.6 wt % and CPC-Ag 1.0 wt % cements, measured by the AES, corresponds to the average values of 25 and 43 µg/L, respectively, reaching a plateau after 15 days.

The results of antibacterial tests prove the inhibitory effect towards pathogenic *Escherichia coli* for both the CPC-Ag 0.6 wt % and the CPC-Ag 1.0 wt % cements, better performances being observed for cement containing a higher Ag-content (1.0 wt %).

## Figures and Tables

**Table 1:** Diameter of inhibition zones at 24 h and 48 h.

Incubation Period, h	Average Diameter of Inhibition Zone, mm		
	CPC-Ag 0 wt %	CPC-Ag 0.6 wt %	CPC-Ag 1.0 wt %
24	0	1.10 ± 0.13	4.90 ± 0.17
48	0	2.40 ± 0.10	5.70 ± 0.21

## Supplementary Materials

Supplementary materials can be accessed at: http://www.mdpi.com/2079-4983/7/2/10/s1.
