# Supplementary material for: Silver-Doped Calcium Phosphate Bone Cements with Antibacterial Properties
Source: J Funct Biomater. 2016 Apr 18;7(2):10. doi: 10.3390/jfb7020010 (PMC4932467; doi:10.3390/jfb7020010)
Supplement: Supplementary file 1 [file jfb-07-00010-s001.pdf]

Journal of
Functional Biomaterials

Supplementary Materials

FTIR Measurement

We have performed FTIR measurement (Nicolet Avatar 330 FTIR spectrometer, England) before and after silver-ion release from cement samples into the TRIS-HCl buffer solution. The cement samples were mixed in a ratio of 1 mg of sample with 300 mg of KBr powder followed by compacting those into a thin pellet in a stainless steel die with 1 cm inner diameter. FTIR data were recorded over the range of 4000 to 400 cm^{-1} with 128 scans.

FTIR spectra of CPC-Ag 0 wt %, CPC-Ag 0.6 wt % and CPC-Ag 1.0 wt % cement samples after 24 h of hardening process are presented on Figure S1. For all investigated cement systems, adsorbed water band was relatively wide, from about 3600 to 2600 cm^{-1}, with an explicit peak at 3570 cm^{-1}. CO_3^{2-} group exhibited weak peak at 2359 cm^{-1} and in the range between 1460 and 1530 cm^{-1}. PO_4^{3-} group vibration bands were observed in the range between 1000 and 1156 cm^{-1}, at 964 cm^{-1}, at 553–610 cm^{-1}, and at 470 cm^{-1}, which is characteristic of β-TCP [s1]. For both CPC-Ag 0.6 wt % and CPC-Ag 1.0 wt % cement samples, HPO_4^{2-} group was detected at 875 cm^{-1} that confirms the appearance of the new DCPD phase. Furthermore, the peaks at ~800 and 1650 cm^{-1}, easily visible for the cement samples, were attributed to the bond vibration of PO_3^- [s2]. Therefore, it can be concluded that $CaAg(PO_3)_3$ was present in both CPC-Ag 0.6 wt% and CPC-Ag 1.0 wt% cement samples.

FTIR spectra of the CPC-Ag 1.0 wt% after 3 and 14 days of silver-ion release test into the TRIS-HCl buffer solution are shown in Figure S2. No $CaAg(PO_3)_3$ product was detected after 14 days, which is proven by the PO_3^- peaks, disappearing at ~800 and 1650 cm^{-1}.

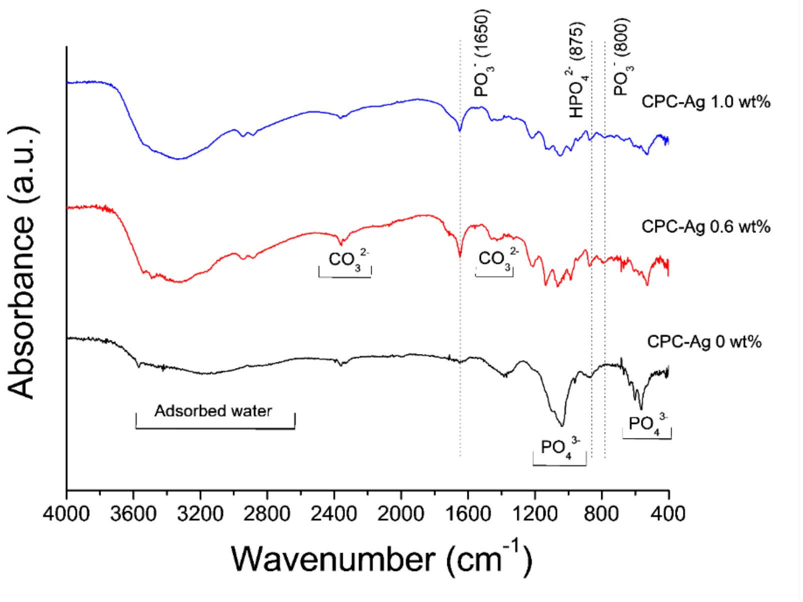

Figure S1. FTIR spectra of CPC-Ag 0 wt %, CPC-Ag 0.6 wt % and CPC-Ag 1.0 wt % cement samples after 24 h of hardening process.

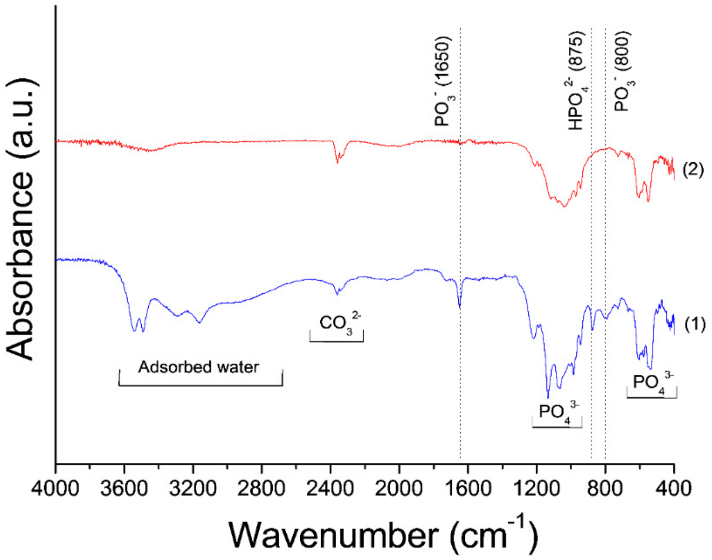

Figure S2. FTIR spectra of the CPC-Ag 1.0 wt % after 3 (1) and 14 (2) days of silver-ion release test into the TRIS-HCl buffer solution.

References

s1 Berzina-Cimdina, L.; Borodajenko, N. Research of calcium phosphates using fourier transform infrared spectroscopy, infrared spectroscopy. In *Materials Science, Engineering and Technology*; Theophile, T., Ed.; InTech: Rijeka, Croatia, 2012; pp. 123–148.

s2 Bozetine, I.; Boukennous, Y.; Trari, M.; Moudir, N. Synthesis and characterization of orthophosphate silver powders. *Energy Procedia* **2013**, *36*, 1158–1167.

© 2016 by the authors; licensee MDPI, Basel, Switzerland. This article is an open access article distributed under the terms and conditions of the Creative Commons by Attribution (CC-BY) license (http://creativecommons.org/licenses/by/4.0/).
